# DING Proteins from Phylogenetically Different Species Share High Degrees of Sequence and Structure Homology and Block Transcription of HIV-1 LTR Promoter

**DOI:** 10.1371/journal.pone.0069623

**Published:** 2013-08-06

**Authors:** Rakhee Sachdeva, Nune Darbinian, Kamel Khalili, Shohreh Amini, Daniel Gonzalez, Ahmed Djeghader, Eric Chabriére, Andrew Suh, Ken Scott, Malgorzata Simm

**Affiliations:** 1 Molecular Virology Division, St. Luke's-Roosevelt Institute for Health Sciences/Columbia University, New York, New York, United States of America; 2 Department of Neuroscience, Temple University School of Medicine, Philadelphia, Pennsylvania, United States of America; 3 Department of Biology, College of Science and Technology, Temple University, Philadelphia, Pennsylvania, United States of America; 4 Enzymologie Structurale, Université de la Méditerranée, Faculté de Médecine, Marseille, France; 5 School of Biological Sciences, University of Auckland, Auckland, New Zealand; National Institute of Infectious Diseases, Japan

## Abstract

Independent research groups reported that DING protein homologues isolated from bacterial, plant and human cells demonstrate the anti-HIV-1 activity. This might indicate that diverse organisms utilize a DING-mediated broad-range protective innate immunity response to pathogen invasion, and that this mechanism is effective also against HIV-1. We performed structural analyses and evaluated the anti-HIV-1 activity for four DING protein homologues isolated from different species. Our data show that bacterial PfluDING, plant p38SJ (pDING), human phosphate binding protein (HPBP) and human extracellular DING from CD4 T cells (X-DING-CD4) share high degrees of structure and sequence homology. According to earlier reports on the anti-HIV-1 activity of pDING and X-DING-CD4, other members of this protein family from bacteria and humans were able to block transcription of HIV-1 and replication of virus in cell based assays. The efficacy studies for DING-mediated HIV-1 LTR and HIV-1 replication blocking activity showed that the LTR transcription inhibitory concentration 50 (IC_50_) values ranged from 0.052–0.449 ng/ml; and the HIV-1 replication IC_50_ values ranged from 0.075–0.311 ng/ml. Treatment of cells with DING protein alters the interaction between p65-NF-κB and HIV-1 LTR. Our data suggest that DING proteins may be part of an innate immunity defense against pathogen invasion; the conserved structure and activity makes them appealing candidates for development of a novel therapeutics targeting HIV-1 transcription.

## Introduction

DING proteins were recently clustered into a new group of highly conserved proteins found in prokaryotes and eukaryotes throughout the plant and animal kingdoms [1], [2], [3], [4], [5], [6], [7]. Phylogenetically, the DING proteins belong to the superfamily of phosphate-binding proteins (PBP) which comprises also PstS and alkaline phosphatase (AP), both exclusively found in prokaryotes [8]. To date, more than fifty different DING proteins have been reported in all kingdoms of life [9]; however, their complete genomic structure and chromosomal assignment in humans is still unknown.

Structural studies on the human phosphate binding protein (HPBP) and the DING protein from *Pseudomonas fluorescens* (PfluDING) revealed interesting details about their configuration [10], [11]. Similarly to the PstS, the DING proteins are formed by two globular domains linked together by a flexible hinge allowing a “Venus flytrap” movement [12]. However, the structural differences on four external loops and presence of two disulfide bridges in DINGs stand them apart from other PBPs and suggest an autonomous class of proteins [12]. The phosphate molecule in the DING protein resides in the vicinity of the binding cleft formed by the two globular domains. The binding of this phosphate ion occurs between four conserved residues in each globular domain, that form a complex network of 12 hydrogen bonds [11]. It has been shown that presence of an aspartic acid (D62) in this phosphate-binding pocket is important for the distinction between the phosphate and other closely-related ions such as sulfate or arsenate [13], [14].

The biology of eukaryotic DING proteins as a group remains to be defined, but most DING proteins isolated from eukaryotes were related to a broad range of disorders and biological processes [1], [2], [6], [7], [15], [16], [17]. For example, the synovial stimulatory protein (SSP) has the capacity to induce proliferation of the peripheral blood T cells in patients with rheumatoid arthritis [6]. Another DING protein, the steroidogenesis-Inducing protein (SIP) has mitogenic activity toward the ovarian epithelium and might be involved in the etiology of ovarian cancer [18]. In contrast, a recent report by Hendriks et *al* indicated that dysfunction of the newly-characterized bovine DING homologue, the gonadotropin surge-Inhibiting/attenuating factor (GnSIF/AF) could lead to polycystic ovarian syndrome [15]. The crystal adhesion inhibitor (CAI), found in monkey and in human renal epithelial cells, has been shown to inhibit the growth of kidney stones [16]. Finally, several members of the DING family inhibit HIV-1 replication through blockage of the LTR transcriptional activity [4], [7], [19], [20]. This comprises HPBP from human plasma [19], X-DING-CD4 from CD4+ T cells [7], [21] and p27SJ (and its full-length form, p38SJ) from the medicinal plant *Hypericum perforatum* [4], [20].

It has been shown that primary target for X-DING-CD4 in blocking HIV-1 activity was NF-κB [22], [23] whereas p38SJ, called hereafter pDING, exhibits the ability to interact with C/EBPβ and Tat, and dephosphorylates the CTD domain of the RNA polymerase II. All these interactions can participate in anti-HIV-1 activity of DING proteins [4], [20], [24]. Subsequent studies showed the ability of pDING and X-DING-CD4 in regulating various cellular genes including MCP-1 and IL-8 by either C/EBPβ- and/or NF-κB-dependent pathways [25], [26]. In addition to the *in vitro* studies, the clinical evaluations indicated that HIV-infected patients have an increased concentration of DING proteins [27], and the increased expression of the X-DING-CD4 mRNA correlates with the cellular restriction of HIV-1 replication in human macrophages [21].

The NF-κB system is involved in the immediate signaling mechanisms of the innate immunity responses against infecting pathogens, including HIV-1 [28]. Activation of NF-κB-dependent transcription of cytokines and other mediators of inflammation exerts protective anti-microbial function. Transcription of several viruses such as Herpes Simplex Virus (HSV), Simian Virus 40 (SV40) and HIV-1 depends on NF-κB signaling [29]. The anti-NF-κB activity of DING proteins is of particular interest in the design of novel anti-viral therapies.

The conserved nature and anti-HIV-1 properties of these DING homologues indicate their function as innate immunity effector molecules and highly recommend consideration of DING proteins as a potential antiretroviral. With this in mind, we performed structural analyses and anti-HIV-1 activity studies for four DING homologues isolated from cells of *Pseudomonas fluorescens* (PfluDING), *Hypericum perforatum* (pDING) and *Homo sapiens* (HPBP and X-DING-CD4). To date, HPBP represent the only DING protein for which a dose response and cytotoxicity assays have been performed [19]. We applied cell-based assays to probe the therapeutic efficacy of DING proteins to block the HIV-1 LTR and virus replication, and calculated the inhibitory concentration 50 (IC_50_). Based on the previous reports showing that the human X-DING-CD4 blocked the p50/p65 NF-κB/HIV-1 LTR binding [22], [30], [31], we employed a chromatin immunoprecipitation (ChIP) assay to observe the recruitment of p50/p65 NF-κB dimer to HIV-1 LTR promoter in cells exposed to treatment by all tested DING homologues.

## Materials and Methods

The studies reported here using human peripheral blood lymphocytes were granted exempt status by the St. Luke's-Roosevelt Institutional Review Board under qualifications listed in section 45.101 (b) (4).

### Cell cultures and reagents

Human astrocytoma U87 MG and MAGI-CCR-5 [32] cell lines were obtained from the American Type Culture Collection (ATCC, Manassas, VA, USA). The 1G5 cells stably expressing a luciferase gene driven by the HIV-1 long terminal promoter (LTR) [33] were obtained through the NIH AIDS Research and Reagent Program Division of AIDS, NIAID. The U87 MG and MAGI-CCR-5 cells were maintained in Dulbecco's Modified Eagle's Medium (DMEM) supplemented with 10% fetal bovine serum (FBS; Life Technologies, Inc.), antibiotics (100 U/ml penicillin and 10 mg/ml streptomycin) and glutamate, while the 1G5 cells were cultured in RPMI 1640 (Sigma) supplemented with 5% FBS, antibiotics and glutamate. Peripheral blood lymphocytes (PBLs) were obtained by elutriation from the whole blood of healthy, HIV-1 negative volunteers. Before the experiment, cells were stimulated for 2 days in Dulbecco's modified Eagle medium (DMEM) supplemented with 10% fetal bovine serum (FBS), phytohemaglutinin (PHA, 5 μg/ml; Sigma), interleukin 2 (10 U/ml; R&D Systems), antibiotics and glutamate. Subsequently, cells were cultured without PHA. All cell cultures were incubated at 37°C in a 7% CO_2_ 95% air-humidified incubator.

### DING proteins

The detailed information referring to the isolation and purification of X-DING-CD4, HPBP and pDING was previously reported [4], [7], [10], [34]. PfluDING protein was used in the form of a site-specific mutation (S32G), altering one of the phosphate-binding residues, and resulting in a protein purified by the same method as the wild-type protein [34]. For evaluations of the biological activity, all four DING homologues were dialyzed against 10 mM Tris-HCl, pH 8.0 using benzoylated cellulose tubing with an MW cut-off of 1.2 kDa (Sigma). Subsequently the dialyzed material was concentrated by lyophilization and stored at 4°C.

### Tertiary structure prediction

Sequences of DING proteins (NCBI Accession Number: AAW57408, ADT62916, YP_002872202.1 and P85173.1 for pDING, X-DING-CD4, PfluDING and HPBP) have been aligned using ClustalW 2.1 [35]. The structure of X-DING-CD4 and pDING was related to sequences of HPBP and PfluDING as a template. Structure alignment was performed by MODELLER 9.11 software [36]. Heteroatoms of pdb files have been removed except the phosphate molecule on the PfluDING structure. The 3D structures were validated using RAMPAGE software [37]. Pymol was used to represent DING protein structures [38].

### SDS PAGE

A total of 50 ng of each DING protein sample was resolved by SDS-PAGE and transferred to supported nitrocellulose membranes as described previously [4]. Proteins were visualized by Coomassie brilliant blue or by western blot using rabbit polyclonal antibody to pDING with the enhanced chemiluminescence detection system (Amersham Pharmacia). Antibody specific for pDING (anti-p27SJ rabbit polyclonal antibody) was obtained from Lampire Biological Laboratories, Inc. Pipersville, PA.

### ChIP assay

The U87 MG cells were transfected using the FuGENE 6 transfection reagent (Roche). Briefly, 1×10^6^ cells were cultured overnight in 100 mm plates. Subsequently, cells were transfected with 1 μg of pGL3-Luc LTR plasmid in the presence of 200 ng/ml of each DING protein, respectively. The pGL3-Luc LTR plasmid used for the chromatin immunoprecipitation (ChIP) assay was generated from the HIV-1 LTR (−374/+43bp) DNA fragment cloned into the pGL3-basic vector (Promega), as we described before [4]. At 48 hours post-transfection, proteins were cross-linked with 1% formaldehyde for 10 min at 37°C; then cells were washed twice in ice-cold PBS supplemented with 1 mM phenylmethylsulfonyl fluoride (PMSF), 1 μg/ml aprotinin and 1 μg/ml pepstatin A, and pelleted for 4 min at 2000 rpm at 4°C. The cell pellets were lysed in 200 μl of lysis buffer (50 mM Tris-HCl, pH 7.4, 150 mM NaCl, 0.1% Nonidet P-40 and protease inhibitors as above) for 10 min on ice. Subsequently, cell lysates were sonicated to shear the genomic DNA to fragments between 200 and 1000 bp, cleared by centrifugation for 10 min at 13,000 rpm at 4°C, and the supernatant was diluted 10-fold in ChIP dilution buffer (0.01% SDS, 1.1% Triton X-100, 1.2 mM EDTA, 16.7 mM Tris-HCl, pH 8.1, 167 mM NaCl). To remove the nonspecific background, all protein extracts were pre-cleared with 80 μl of salmon sperm DNA/protein A agarose-50% slurry for 30 min at 4°C. For the IP reaction, 250 μg of each protein sample was incubated overnight at 4°C with 2 μl of anti-p65 NF-κB (F-6) antibody (Santa Cruz Biotechnology) or 2 μl of control normal mouse serum (Pierce Chemical). The IP complexes were precipitated by addition of 60 μl of salmon sperm DNA/protein A agarose slurry for 1 hour at 4°C and collected by centrifugation at 1000 rpm at 4°C. Following washing for 3–5 min at room temperature (RT) with 1 ml of low salt immune complex wash buffer (0.1% SDS, 1% Triton X-100, 2 mM EDTA, 20 mM Tris-HCl, pH 8.1, 150 mM NaCl), high salt immune complex wash buffer (0.1% SDS, 1% Triton X-100, 2 mM EDTA, 20 mM Tris-HCl, pH 8.1, 500 mM NaCl), LiCl immune complex wash buffer (0.25 M LiCl, 1% Nonidet P-40, 1% sodium deoxycholate, 1 mM EDTA, 10 mM Tris-HCl, pH 8.1), and 1X TE buffer, the IP complexes were eluted for 15 min with the elution buffer (Tris-HCl, 1 mM EDTA, pH 8.0). The antibody/protein/LTR crosslinking was reversed by incubation with 8 μl 5 M NaCl at 65°C for 4 hours. Proteins were digested in 10 μl of 0.5 M EDTA, 20 μl 1 M Tris-HCl, pH 6.5 and 2 μl of 10 mg/ml Proteinase K for 1 hour at 45°C and DNA was recovered by phenol/chloroform extraction and ethanol precipitation. The immunoprecipitated HIV-1 LTR was detected by PCR amplification with primers flanking the NF-κB region as follows: sense 5′-(-374)-TTTGCAGAACTACACACCAGGGC C-(-351)-3′ and antisense 5′-(-75)-CTCCCTGGAAAGTCCCCAGCGGAA)-(-98)-3′. The DNA fragments were analyzed in 2% agarose gel and subjected to band densitometry.

### Rapid Suppression Assay (RSA)

RSA was performed essentially as described before [30]. Briefly, 1G5 cells, stably transfected with an inducible luciferase gene driven by HIV-1 LTR [33], were washed in PBS, and resuspended in hybridoma medium to a concentration of 5×10^6^ cells/ml. For control titration curves, 100 μl aliquots of 1G5 cells were supplemented with increasing amounts of DING proteins, control C3 Peptide P16 or medium alone, brought to a final volume of 200 μl and incubated for 3 hours at 37°C. The C3 Peptide P16 is derived from the C3d component of serum complement; it regulates B cells, but not T cells, through interacting with the gp140 C3d receptor (CR2) [39]. Subsequently, cells were induced with phorbol myristate acetate (PMA; Sigma; 5 ng/ml). Two control tubes containing 1G5 cells were resuspended in hybridoma medium (Invitrogen), with or without PMA. Three hours later cells were lysed using reporter lysis buffer (Promega). Luciferase protein expression was measured according to the manufacturer's protocol. All data were normalized by total protein concentration measured by protein assay (Bio-Rad). The HIV-LTR inhibition values were established from the formula: [(LUC_1_-LUC_0_)x100/Z]-100; where LUC_1_ is the value obtained from PMA-induced cells treated with a specific dilution of DING protein, and LUC_0_ is the basal value obtained from uninduced, untreated cells; Z is the absolute luciferase induction by PMA calculated as Z = LUC_max_-LUC_0,_ where LUC_max_ is the value of 100% luciferase expression in PMA-induced, untreated cells.

### MAGI assay

The MAGI assay [32] was performed with modifications as described before [7]. Briefly, MAGI-CCR-5 cells were seeded 24 hours prior to assay in a 96-well plate (Costar Scientific) at 6.2×10^3^ cells per well in DMEM supplemented with 10% fetal bovine serum, antibiotics and glutamate (all from Sigma). Subsequently, cells were exposed to DING protein or control C3 Peptide P16 treatments. Twenty four hours later cells were infected with 0.1 pg/cell NL4-3 HIV-1 isolate [40]. Replication of virus was evaluated 48 hours later after fixation of cells with 1% formaldehyde and 0.2% glutaraldehyde in PBS. The expression of β-galactosidase was visualized by 50 min exposure to X-GAL (5-bromo-4-chloro-3-indolyl-β-D-galactopyranoside), 0.4 mg/ml 2 mM MgCl_2_, 4 mM potassium ferricyanide at 37°C. After enumeration of the infected (blue) cells, all cells were lysed and subjected to the protein assay (Bio-Rad) to establish total protein sample concentration, and all data were normalized by total protein concentration. The values for inhibition of HIV-1 replication were calculated based on the formula [(R_1_–R_0_)x100/Z]-100, where R_1_ is the value obtained from cells infected by HIV-1 and treated with a specific dilution of DING protein and R_0_ is the basal value obtained from the uninfected, untreated cells; Z is the absolute HIV-1 replication value calculated as Z = R_max_-R_0,_ where R_max_ is the value representing 100% of HIV-1 replication in the untreated cells.

The IC_50_ values were calculated from titration curves using two adjoining dilutions for each DING protein that showed inhibition close to 50% inhibition of LTR (RSA) or HIV-1 replication (MAGI).

### Evaluation of DING protein-mediated antiviral activity in primary human cells

3×10^6^ PBLs/well in a 24-well plate (Costar Scientific) were cultured in 1 ml RPMI medium supplemented with antibiotics, glutamate and 1 μg/ml of each DING protein, respectively. Six hours after the initial exposure to the DING treatments, the culture medium was supplemented with 3%/Vol FBS. One day after treatment, cells were infected with 0.01 pg/cell NL4-3 HIV-1 isolate [40] and cultured as described above, except that the concentration of FBS was adjusted to 5%/Vol. The experimental control consisted of HIV-1-infected but untreated PBLs. Replication of virus was evaluated at five and seven days after infection by Elisa assay of the intracellular HIV-1 p24 core antigen (Perkin Elmer). The viability of cells was assessed by the dye exclusion method [41] at 1, 2, 3, 5 and 7 days after DING treatments.

## Results

### The structure of DING proteins

**Figure 1:**
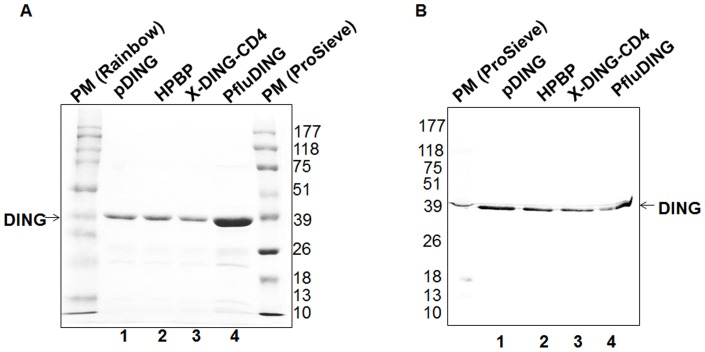
Testing the mobility of DING protein variants in SDS-PAGE. (A) Coomassie brilliant blue staining of SDS PAGE gel. The first and last lanes correspond to the molecular weight marker (respectively Rainbow and ProSieve). Lanes 1–4 correspond to pDING, HPBP, X-DING-CD4, and PfluDING. (B) Western Blot of DING proteins. The first lane corresponds to molecular weight marker (ProSieve), bands lower than 39 kDa in lane 4 indicate degradation products of PfluDING.

**Figure 2:**
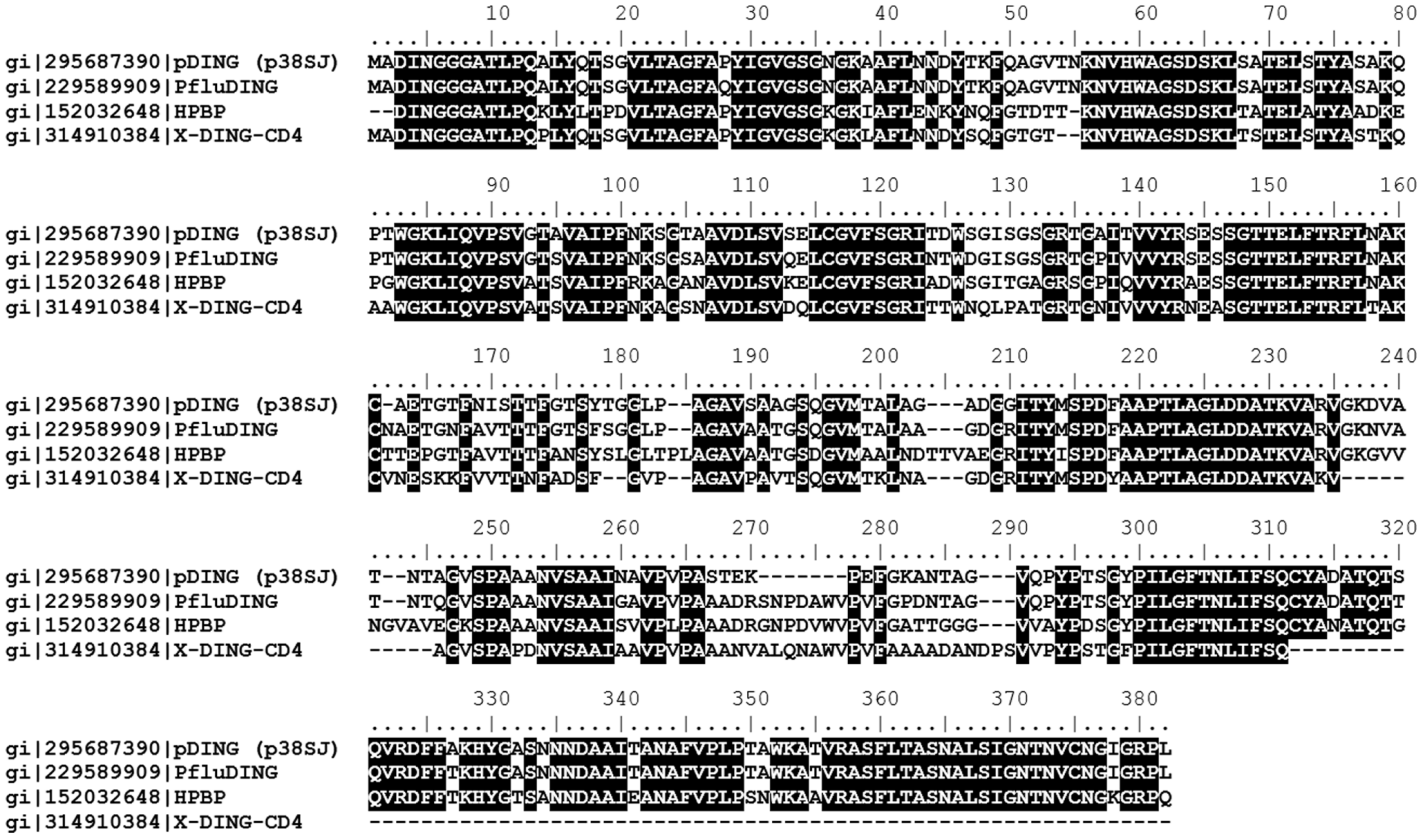
The amino acid alignment for pDING, PfluDING, X-DING-CD4 and HPBP proteins. Accession numbers for each protein are indicated between bars, identical residues are highlighted with black. The sequence alignment was done using Bioedit software.

As shown in Figure 1A, the four DING proteins migrated with similar mobility of about 39 kDa. All of them are recognized by antibody generated to pDING (Fig. 1B), and that seems to be due to the high sequence identity shared by the four DING homologues (Fig. 2).

**Figure 3:**
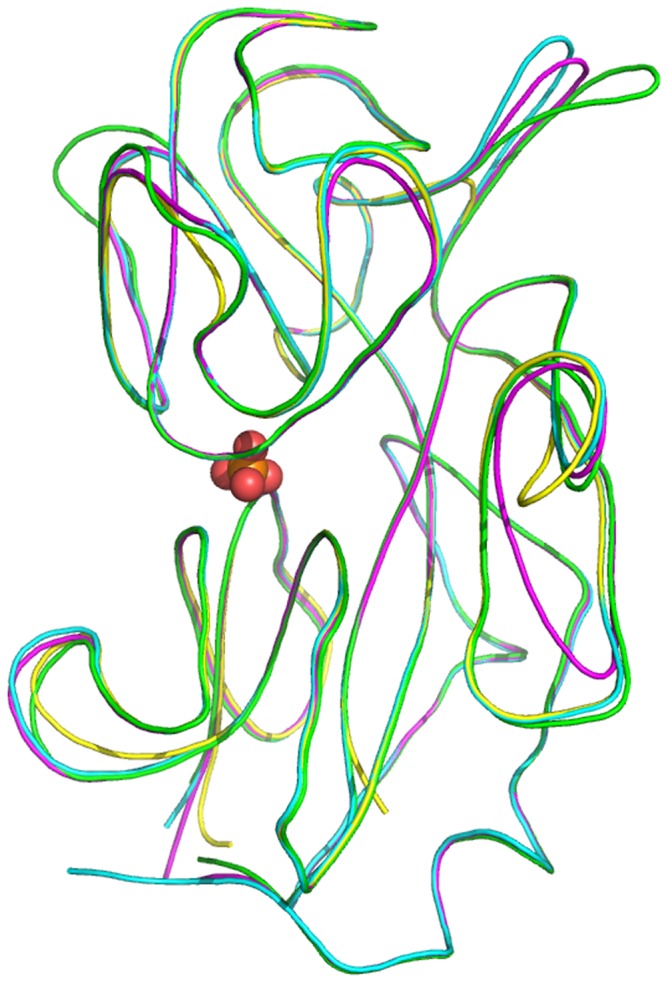
Structure superposition of the four different DING proteins. Structure alignment for X-DING-CD4 and pDING was modeled into to HPBP and PfluDING X-ray structures. The backbone of PfluDING (pdb:2Q9T), HPBP (pdb:2V3Q), p38SJ (pDING) and X-DING-CD4 is shown in green, cyan, magenta and yellow, respectively. The phosphate molecule is marked by sphere.

The only existing crystal structures of DING proteins were derived from the HPBP [10] and the bacterial PfluDING [11]. We used this information for prediction of the X-DING-CD4 and pDING structures (Fig. 3). Our analysis showed that all tested proteins share the same topology with closely-similar structures. The protein backbone is perfectly superimposed between the four DING proteins, particularly in the zone implicated in phosphate binding (Fig. 3, sphere). The structural difference was noted only in the length of protuberant loops, which generated a visible disparity in some regions between the four DING proteins.

### Testing the anti-HIV-1 therapeutic utility of DING protein variants

Independent studies demonstrated that phylogenetically-distinctive DING proteins might have similar HIV-1 blocking activity [4], [7], [19]. To better understand the specific anti-viral characteristics of these DING proteins, we compared their antiviral potential in a uniform experimental setting. We utilized the rapid suppression assay (RSA) [30] which tests directly the inhibition of HIV-1 transcription, the MAGI assay which tests the inhibition of HIV-1 replication in a single cycle infection [32], and evaluated the DING – mediated inhibition of HIV-1 infection in human PBLs.

**Figure 4:**
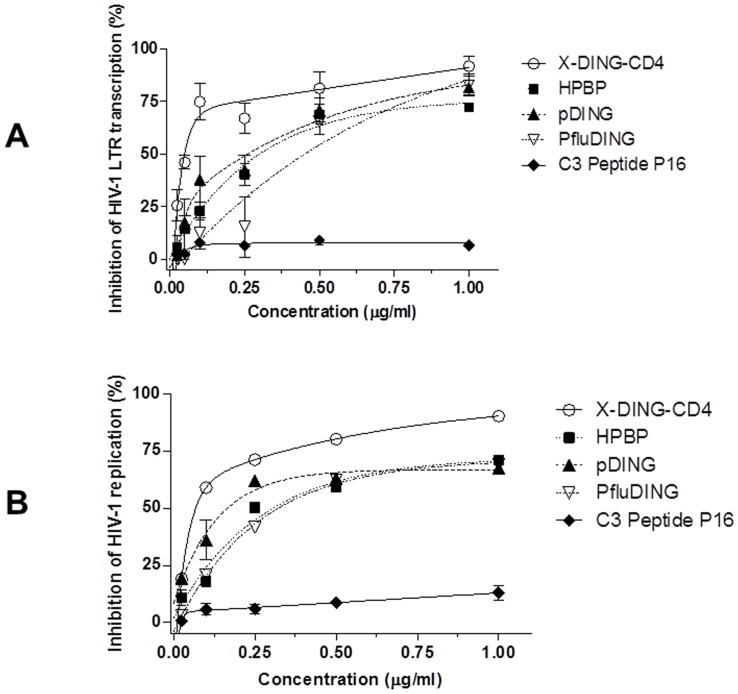
Evaluation of pDING, PfluDING, X-DING-CD4 and HPBP therapeutic efficacy against HIV-1. Dose versus response was calculated by GraphPad Prism version 5.00 for Windows. The efficacy of selected DING proteins to block HIV-1 LTR transcription was measured by RSA (A); and to block HIV-1 replication was measured by MAGI (B). The C3 peptide P16 was used as a negative control. The results are representative of at least three separate experiments.

As shown in Fig. 4A, HPBP, pDING, PfluDING and X-DING-CD4 blocked transcription of HIV-1 LTR by 75% at concentrations ranging from 0.1–1 μg/ml. The HIV-1 LTR IC_50_ for human X-DING-CD4 and HPBP was 52 ng/ml and 449 ng/ml, respectively (Fig. 4A and Table 1). The IC_50_ values for plant pDING and bacterial PfluDING were 160 ng/ml and 254 ng/ml, in that order (Fig. 4A and Table 1). Treatment of cells with control C3 Peptide P16 had only a minor effect on HIV-1 LTR activity, blocking its expression by 4–9%.

**Table 1:** The comparison of the IC_50_ values acquired from dose versus response evaluations in MAGI and RSA.

Treatment	IC_50_ (μg/ml)	
	MAGI	RSA
X-DING-CD4	0.075	0.052
HPBP	0.150	0.449
pDING	0.101	0.160
*Pflu*DING-S32G	0.311	0.254

Similarly to the evaluations of DING-mediated inhibition of HIV-1 LTR transcription, the MAGI assay showed that replication of HIV-1 was also blocked by all tested DING protein homologues (Fig. 4B). This data suggested that successful inhibition of the LTR transcription also interrupted the subsequent stages of the HIV-1 life cycle. The IC_50_ values ranged from 75 ng/ml for X-DING-CD4 to 311 ng/ml for PfluDING (Fig. 4B and Table 1). The IC_50_ for HPBP and pDING was calculated as 150 ng/ml and 101 ng/ml, respectively (Fig. 4B and Table 1). The control C3 Peptide P16 had only a minor effect on HIV-1 replication, with 11% inhibition at the highest dose of 1 μg/ml.

**Figure 5:**
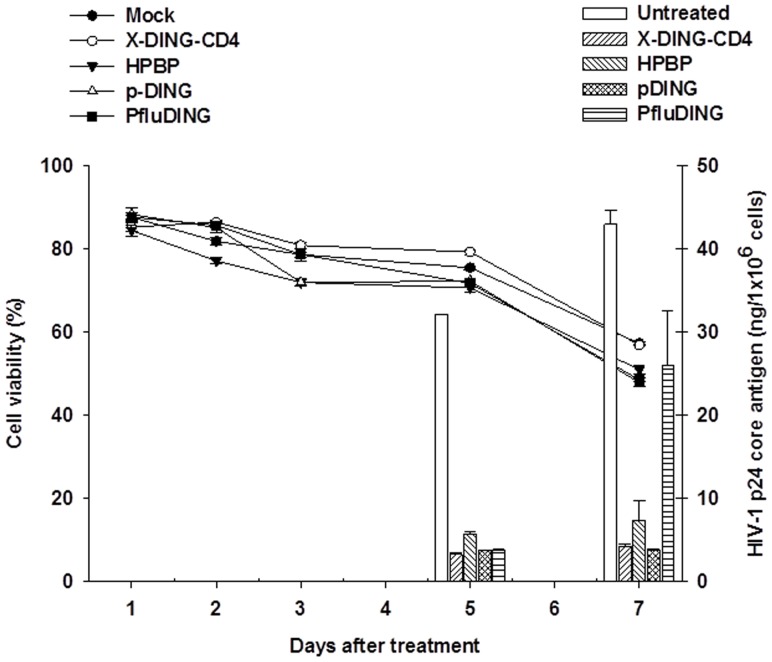
Human, plant and bacterial DING proteins block replication of HIV-1 in human PBLs. Replication of HIV-1 was assessed by measurements of HIV-1 p24 core antigen (bars, right axis) and the viability of cells was established by the dye exclusion method (lines, left axis). The results are representative of at least three separate measurements.

The RSA and MAGI data reflected the direct effect of DING proteins on HIV-1 LTR transcription and the replication of virus in a single-cycle infection. To study DING-mediated restriction of virus in the normal course of infection, we subjected HIV-1-infected PBLs to DING treatments and measured replication of virus five and seven days later, assessing the intracellular levels of p24 core protein. As shown in Fig. 5 (bars – right axis), all DING homologues blocked replication of HIV-1. Five days after infection, the intracellular p24 core protein was lower by 6 to 10-fold in DING-treated samples as compared to the untreated control. Seven days after infection, the X-DING-CD4 and pDING treatments reduced replication of HIV-1 by 11-fold, HPBP by 6-fold and PfluDING by 1.7-fold. It is important to note that throughout the course of this experiment, the viability of cells treated with DING protein variants was comparable to the untreated sample, thus alleviating concerns of treatment-induced cytotoxicity (Fig. 5 lines – left axis).

The HIV-1 LTR transcription is not targeted by any of the currently-available antiviral therapies. Although the complete mechanism of DING proteins is yet to be defined, the published data indicate that X-DING-CD4, HPBP and pDING block the HIV-1 at the level of LTR transcription [4], [7], [19], [22], [24], [31], and current investigation confirmed this LTR-blocking activity also for the bacterial PfluDING variant (Fig. 4A).

**Figure 6:**
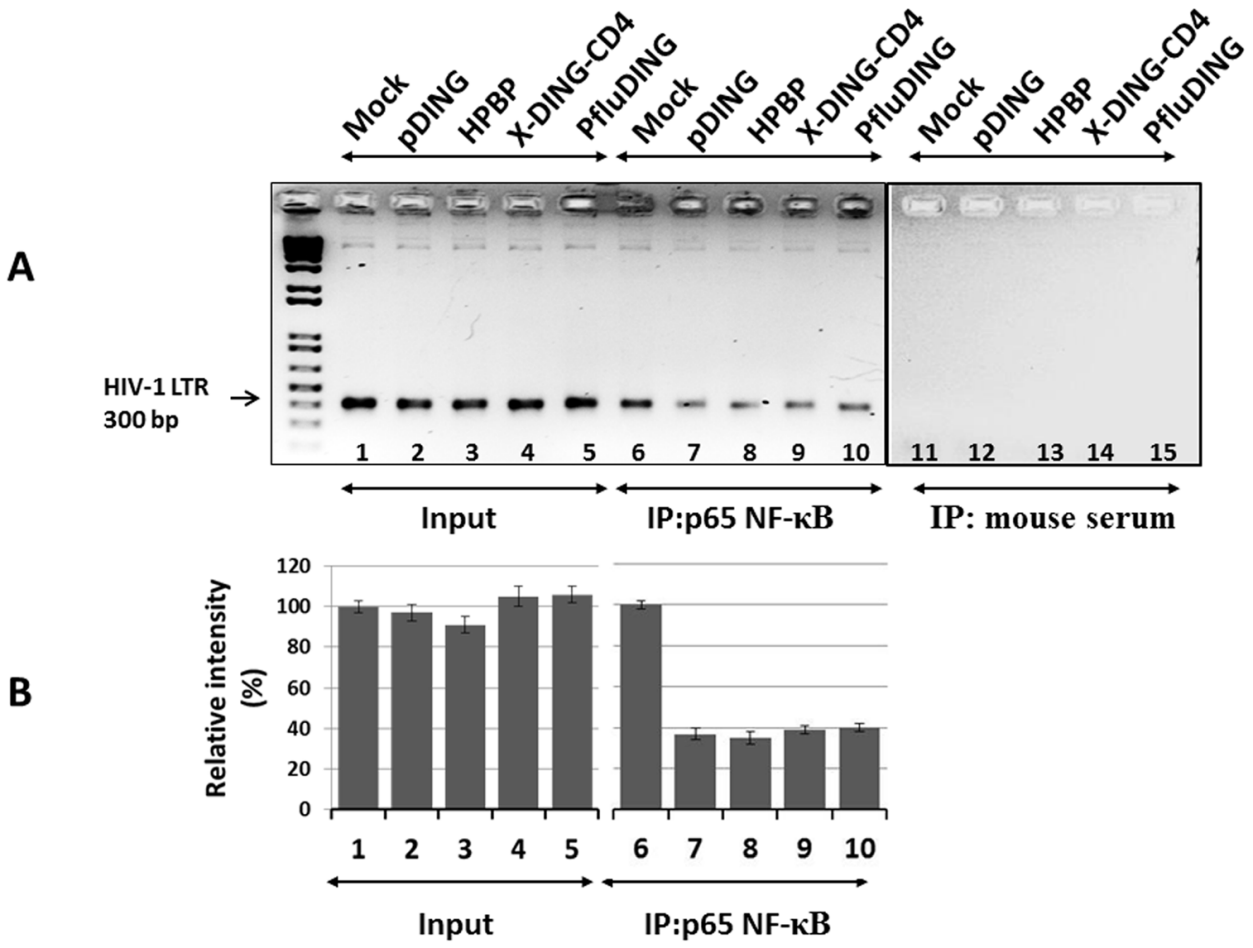
Human, plant and bacterial DING proteins block the recruitment of p65-NF-κB to HIV-1 LTR. (A) The p65 NF-κB occupancy on HIV-1 LTR was tested in U87MG cells exposed to four DING proteins. The 300 bp HIV-1 LTR fragment was amplified with a pair of primers used for detection of NF-κB binding. (B) the optical density of PCR amplicons was tested by the Adobe Photoshop software and ImageJ analysis Program. The (input) represents total amount of HIV-1 LTR DNA before the immunoprecipitation; (IP:p65 NF-κB) – represents the amount of p65 NF-κB/LTR complex in cells treated or not (Mock) with each DING protein. (IP: mouse serum) – indicates immunoprecipitation with a non-specific serum. Data were analyzed using Excel software. The results are representative of at least two separate experiments.

In light of earlier results pointing to the potential involvement of NF-κB in suppression of HIV-1 by X-DING-CD4 [22], [30], [31], we focused our attention on examining whether the other DING counterparts can interfere with binding of NF-κB to the LTR DNA sequence. To this end, the human astrocytic cell line, U-87MG, transduced with HIV-1 LTR plasmid, was exposed to each DING protein variant at a concentration of 200 ng/ml; this concentration exceeded the IC_50_ dose for the inhibition of HIV-1 LTR transcription and replication (Fig. 4A, B and Table 1). As shown in Fig. 6A, the recruitment of p65-NF-κB to the HIV-1 LTR promoter was severely impaired in the presence of each DING protein (lanes 6–10) as compared to the input DNA control (lanes 1–5); the specificity of this reaction was compared to the control mouse serum which was negative for detection of p65-NF-κB/LTR complex (lanes 11–15). Based on the band densitometry analysis (Fig. 6B), the pDING and HPBP blocked the p65-NF-κB/HIV-1 LTR binding by 61.9 and 61.5%, respectively; while the X-DING-CD4 and PfluDING inhibited formation of this complex by 62.9 and 62.3%, in that order. Overall these results confirmed the previously-published observations for the X-DING-CD4- mediated inhibition of p65 NF-κB/LTR binding [22], [31]; it also demonstrated that three other DING protein homologues had similar abilities to block HIV-1 LTR transcription in human cells. Our previous reports indicate that X-DING-CD4 treatment did not affect the p50/p65NF-κB nuclear translocation [22], therefore reduction of p65 NF-κB/DNA binding shown in Fig. 6 is a result of DING-mediated activity and not reduced concentration of nuclear p65 NF-κB.

## Discussion

The DING proteins form an intriguing family of biologically active factors contributing to protective [7], [15], [16], [27], [42], [43] or possibly adverse [1], [6] cellular functions. In this work we selected four distinct members of the DING family to ascertain their structural and functional properties with specific interest in their anti-HIV-1 activity.

The structural studies indicated that four tested DING proteins have highly-conserved protein backbones and differ in the length of the protuberant loops. The perfect superimposition observed in the phosphate-binding cleft confirmed that these proteins have been specifically designed to bind a phosphate ion.

The differences in the length of protuberant loops could be connected to the various physiological functions attributed to DING proteins and may be related to their specific protein/protein interactions (PON1, NF-κB, Tat or C/EBPβ) [4], [20], [22], [25], [26], [27], [44], [45]. Nevertheless, the native function of DING proteins, and particularly pDING, is yet to be determined, but plant DING proteins associate with germin-like proteins (GLPs) [46] known to have multiple functions including pathogen elicitation [47].

As a dose response assay has been performed only for HPBP in a previous study [19], it was worthwhile to determine the IC_50_ values for all tested DING proteins in a single experiment. Our result confirmed those previously obtained for HPBP; the value of 150 ng/ml obtained from this experiment for the inhibition of HIV-1 replication was close to that published by Cherrier et *al* (190 ng/ml) [19]. However, while IC_50_ values obtained for X-DING-CD4, pDING and PfluDING corresponded closely for the RSA and MAGI assays, this was not the case for the HPBP. We think that that it may be due to the instability of this protein (E.C. personal communication), since the value obtained with the MAGI assay was significantly low and close to that obtained by Cherrier *et* *al* [19]. This might also explain the results obtained in primary human cells. Results from this study also permitted the determination of an order of efficiency of DING proteins for HIV-1 inhibition. In fact, all DING proteins do not inhibit HIV-1 similarly. We showed that X-DING-CD4 was the most potent inhibitor of HIV-1 transcription and replication, followed by HPBP and pDING approximately at the same level and finally by PfluDING. This difference of HIV-1 inhibition efficiency is likely to be related to a specific part of the DING sequence (or structure) that needs to be determined. It also might be related to specific posttranslational modifications, absent in bacterial DING. We found both methylated and un-methylated (E68) forms of X-DING-CD4 protein during its purification from cell culture supernatants [7].

Results from NF-κB binding assays indicated that the four tested DING proteins are able to block the formation of the p65-NF-κB/HIV-1 LTR complex. The NF-κB family is composed of five proteins (p50, p52, p65, RelB and c-Rel) that form various complexes of transcription factors involved in inflammation, cell proliferation and immunity [48]. The activation of NF-κB may occur in response to different stimuli, including bacterial and viral infections, and is triggered via different pathways [48], [49]. Out of 15 known NF-κB homo- and heterodimers formed in cells, the p65/p50 NF-κB is most abundant [50], rapidly activated [51] and most importantly, used by HIV-1 during LTR-transactivation [52]. Densitometry analysis showed no significant differences in the rate of inhibition between the four DING proteins that ranged from 61.5 to 62.9%. This result indicates that all tested DING proteins inhibit NF-κB binding to LTR similarly when used at concentrations exceeding the IC_50_ value (200 ng/ml). The discrepancy between the IC values obtained from the efficacy evaluations (RSA and MAGI) and those obtained from ChIP assay could be explained by use of distinct experimental tools. For example, the RSA measures DING-mediated inhibition of the whole LTR promoter, while the ChIP assay is performed upon the LTR probe encompassing only the NF-κB binding sequences. We believe that minor structural differences between DING proteins could impose unique physiological functions upon individual DING variants, such as targeting several events in transcription from the HIV-1 promoter, as reported for pDING [4], [20], [44]. Nonetheless, the inhibition of NF-κB/LTR binding seems to be a common trait for all tested DING variants.

We indicated before that the eukaryotic genes encoding DING proteins might originate from evolutionary conservation [53]. The notable preservation of their structure and function could have its origin in the selective pressure necessary to maintain successful clearance of invading pathogens. Maintenance of these genes throughout the process of evolution was likely related to the retention and development of functions essential to survive the pathogen invasion. These studies provide direct proof for this hypothesis. DING homologues from distinctly diverse organisms retain a structural and functional resemblance. The bacterial, plant and human DING proteins blocked transcription of the HIV-1 LTR promoter in cell-based assays suggesting that these proteins might permeate the cell membrane or interact with a cell-surface receptor in similar ways. The mechanism of X-DING-CD4-cell membrane interactions and downstream effects of these interactions is currently under investigation.

We believe that the LTR-blocking activity is, in part, a consequence of a broad-spectrum DING-mediated mechanism to block pathogen-induced activation of NF-κB-dependent promoters [22], [25], [26]. This function becomes a strategic advantage in the event of HIV disease. Nevertheless, the presence of such proteins in some opportunistic pathogens (like *Pseudomonas*) may interfere with this mechanism of innate immunity. In fact it has been shown that X-DING-CD4 is able to block LPS-mediated induction of NF-κB-dependent IL-8 transcription, and thus interfere with the inflammatory process [25]. That means that it is not to be excluded that bacteria may use their own DING proteins in order to block the host's inflammatory mechanism upon invasion.

Previous study showed the ability of pDING to interfere with other critical events involved in HIV-1 replication that include association of C/EBPβ with the HIV-1 genome, nuclear localization of HIV-1 Tat, and phosphorylation of C-terminal polymerase by pTEF [20]. Based on extensive sequence homology, it is anticipated that the other members of the DING family can also exert activities similar to pDING on the other events that may impact LTR transcription and replication. In earlier studies, we showed that some HIV-1-infected individuals might control virus replication through the induction of X-DING-CD4 gene activity [21], and new studies indicate that expression of X-DING-CD4 mRNA is significantly higher in peripheral blood mononuclear cells (PBMCs) from elite HIV-1 controllers than in AIDS patients or uninfected controls [54]. This alone indicates that X-DING-CD4, and possibly HPBP, function as molecules of the human innate immunity system, while their counterpart, the pDING, may have a similar function in plants. The fact of pDING isolation from plant callus tissue [4] indicates co-localization of this DING protein within the injured tissue caused by pathogenic invasion.

In summary, we conclude that DING proteins form a distinctive group of highly conserved biomolecules with highly redundant properties, some of which are directed to protective anti-microbial function across the species. Four members of this family block HIV-1 transcription and their role in cellular innate immunity responses needs further investigation. DING proteins are also an attractive target for drug development, in particular because none of the existing components of antiretroviral therapy targets HIV-1 transcription.
